# Disulfidptosis-Associated Neurotoxicity Induced by Cadmium Under an Environmentally Relevant Cadmium Exposure Scenario

**DOI:** 10.3390/ijms27146330

**Published:** 2026-07-16

**Authors:** Jingxia Wei, Jinhao Wan, Xinyu Yuan, Tianao Sun, Yongjie Ma, Minglian Pan, Zhanyue Zheng, Yingjie Zhou, Yan Sun

**Affiliations:** 1 School of Public Health, Guilin Medical University, Guilin 541199, China; m18777893571@163.com (J.W.); wjhwio@163.com (J.W.); 18285094298@163.com (X.Y.); 17791635922@163.com (T.S.); mayongjie0606@163.com (Y.M.); pml18278456264@163.com (M.P.); zhengzhanyue999@163.com (Z.Z.); 15221582965@163.com (Y.Z.); 2 Guangxi Key Laboratory of Environmental Exposomics and Entire Lifecycle Health, Guilin Medical University, Guilin 541199, China

**Keywords:** cadmium, disulfidptosis, neurotoxicity, oxidative stress

## Abstract

Cadmium (Cd) is a widespread environmental pollutant associated with neurotoxicity, but its underlying mechanisms remain unclear. Disulfidptosis is a regulated cell death driven by disulfide stress under conditions of impaired cellular reducing capacity. This study investigated the potential involvement of disulfidptosis-associated molecular alterations in Cd-induced neurotoxicity. Male Sprague Dawley (SD) rats were exposed to cadmium chloride (Low-Dose Group: CdCl_2_: 0.036 mg/kg bw; High-Dose Group: CdCl_2_: 3.6 mg/kg bw) by oral gavage for 30 days. Neurobehavioral performance was assessed using the open field test, elevated plus maze, and Morris water maze. Hippocampal ultrastructure, redox-related metabolites, and disulfidptosis-associated genes were analyzed. In addition, bioinformatics analysis was performed by integrating cadmium-related, neurodegenerative disease-related, and disulfidptosis-related genes. The results showed that high-dose Cd exposure impaired locomotor activity, increased anxiety-like behavior, and disrupted spatial learning and memory (*p* < 0.05), accompanied by mitochondrial damage in hippocampal neurons. Bioinformatics analysis identified seven overlapping genes and enrichment of ferroptosis and oxidative phosphorylation pathways. Biochemically, cadmium exposure significantly increased the NADP^+^/NADPH ratio ([Control: 1.07 ± 0.044] vs. [High-dose: 3.80 ± 0.059], *p* < 0.05) and decreased the GSH/GSSG ratio ([Control: 2.80 ± 0.059] vs. [High-dose: 1.14 ± 0.091], *p* < 0.05), indicating severe redox imbalance. At the molecular level, cadmium exposure upregulated SLC7A11 mRNA expression by 1.48 ± 0.12-fold (*p* < 0.01) and SLC3A2 by 1.91 ± 0.55-fold (*p* < 0.05), while downregulating NDUFS1 expression to 0.84 ± 0.01-fold of control levels (*p* < 0.01) in hippocampal tissues. These findings suggest that high-dose Cd exposure induced neurotoxicity is associated with mitochondrial dysfunction, redox imbalance, and disulfidptosis-associated molecular alterations.

## 1. Introduction

Cadmium (Cd) is a persistent environmental contaminant widely distributed in soil, water, and food systems as a result of mining, smelting, and other industrial activities. Cd is recognized as a multi-organ toxicant with a remarkably long biological half-life of approximately 20–30 years in humans [1]. Following chronic exposure through contaminated food, drinking water, cigarette smoke, or occupational settings, Cd accumulates in multiple tissues owing to its poor excretion. Numerous studies have demonstrated that Cd exerts cytotoxic effects in the kidney, liver, lung, cardiovascular system, reproductive organs, bone, and endocrine tissues [2,3]. Experimental and epidemiological studies have shown that Cd toxicity is characterized by excessive reactive oxygen species generation, mitochondrial dysfunction, disruption of calcium homeostasis, DNA damage, endoplasmic reticulum stress, inflammatory activation, and impairment of antioxidant defense systems, ultimately leading to cellular dysfunction and death [4,5].

Due to the fact that neurons have a very high oxygen consumption, abundant mitochondria and limited regenerative capacity, the nervous system is particularly susceptible to Cd [6]. Moreover, neuronal function critically depends on continuous ATP production and tightly regulated redox homeostasis. Cd can penetrate the blood–brain barrier through divalent metal transporters and gradually accumulate in brain tissue [7]. Once inside the central nervous system, Cd promotes oxidative stress, impairs mitochondrial respiration, disturbs calcium signaling, inhibits neurotransmitter release, and activates neuroinflammatory responses, thereby contributing to cognitive dysfunction and neurodegenerative diseases [8].

In recent years, increasing evidence has demonstrated that regulated cell death plays a central role in Cd toxicity. Multiple forms of programmed cell death, including apoptosis, autophagy, pyroptosis, necroptosis, ferroptosis, and cuproptosis, have been implicated in Cd-induced tissue injury [9,10]. These pathways share extensive crosstalk through oxidative stress, mitochondrial dysfunction, and metabolic reprogramming. Disulfidptosis has emerged as a distinct form of regulated cell death characterized by intracellular disulfide stress rather than lipid peroxidation or caspase activation [11].

Recent advances in cell death research have identified disulfidptosis as a distinct form of programmed cell death driven by NADPH depletion and dysregulated cystine metabolism, leading to excessive disulfide bond formation and cytoskeletal collapse [12,13,14]. This mechanism has been primarily described in proliferative cell systems, such as cancer cells, where high expression of the cystine/glutamate antiporter system Xc^−^ renders cells vulnerable to disulfide stress under conditions of impaired glucose metabolism [12]. Neurons are highly dependent on mitochondrial oxidative phosphorylation and continuous NADPH regeneration to maintain redox homeostasis [15]. Therefore, neuronal cells may be particularly vulnerable to disulfide stress when reducing capacity is compromised [16]. Importantly, cadmium is known to disrupt NADPH-generating pathways and glutathione metabolism [17], suggesting that cadmium exposure may create a metabolic context permissive for disulfidptosis-like mechanisms in neural tissues.

Considerable efforts have been devoted to preventing Cd-induced cytotoxicity. Current preventive strategies include reducing environmental exposure, nutritional supplementation with essential trace elements (e.g., selenium and zinc) [18], antioxidant therapy, chelation treatment, and modulation of intracellular redox homeostasis [19]. However, these approaches mainly alleviate oxidative injury rather than targeting the upstream molecular events responsible for Cd-induced cell death. Therefore, identifying novel mechanisms of Cd cytotoxicity may facilitate the development of more effective therapeutic interventions.

Despite accumulating evidence demonstrating that Cd induces oxidative stress and mitochondrial dysfunction, whether disulfidptosis contributes to Cd-induced neurotoxicity remains largely unknown. Furthermore, experimental evidence linking Cd exposure with disulfidptosis-associated molecular alterations in the nervous system is still lacking. Therefore, the present study was designed to investigate whether environmentally relevant Cd exposure induces a disulfidptosis-permissive cellular environment in the hippocampus. By integrating bioinformatics analysis with behavioral assessments, ultrastructural analysis, measurements of redox-related metabolites and disulfidptosis-associated molecular markers, the potential mechanisms of disulfidptosis in Cd-induced neurotoxicity are explored.

## 2. Results

### 2.1. Identification of Disulfidptosis-Related Genes Associated with Cadmium Exposure and Neurodegenerative Disease

Intersection analysis among cadmium-related genes, neurodegenerative disease-related genes, and ten disulfidptosis-associated genes identified seven common genes: NCKAP1, NDUFS1, NDUFS5, NUBPL, RPN1, SLC3A2, and SLC7A11. These genes may represent key molecular targets linking cadmium exposure to neurodegenerative disease through disulfidptosis-associated pathways.

**Figure 1:**
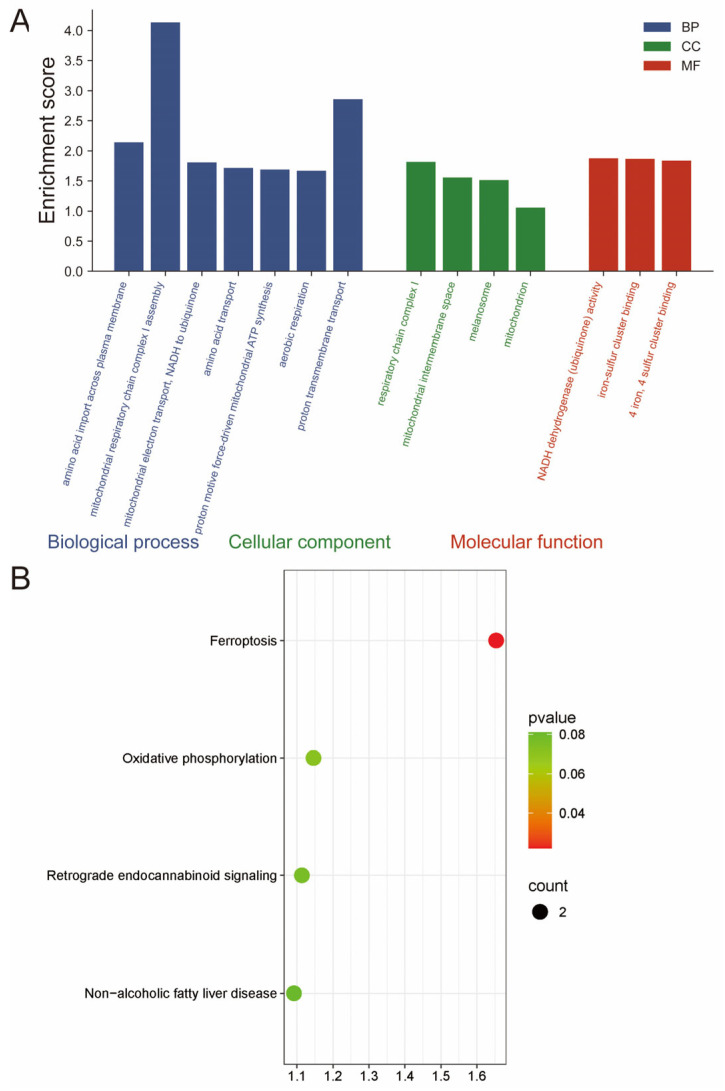
Result of bioinformatics analysis. (**A**) GO analysis of the intersection genes. (**B**) KEGG analysis of the intersection genes.

GO (Gene Ontology) enrichment analysis revealed that the overlapping genes were mainly involved in amino acid import across plasma membrane, mitochondrial respiratory chain complex assembly, mitochondrial electron transport, NADH to ubiquinone, amino acid transport. In the Cellular Component category, these genes were mainly enriched in mitochondrial intermembrane space, respiratory chain complex I, melanosome, mitochondrion. In the Molecular Function category, enrichment was primarily observed in NADH dehydrogenase (ubiquinone) activity, iron–sulfur cluster binding (Figure 1).

KEGG (Kyoto Encyclopedia of Genes and Genomes) pathway analysis demonstrated that the overlapping genes were significantly enriched in pathways associated with Ferroptosis, Oxidative phosphorylation, Retrograde endocannabinoid signaling, Non-alcoholic fatty liver disease (Figure 1).

### 2.2. Cadmium Exposure Altered Neurobehavioral Performance in Rats

**Figure 2:**
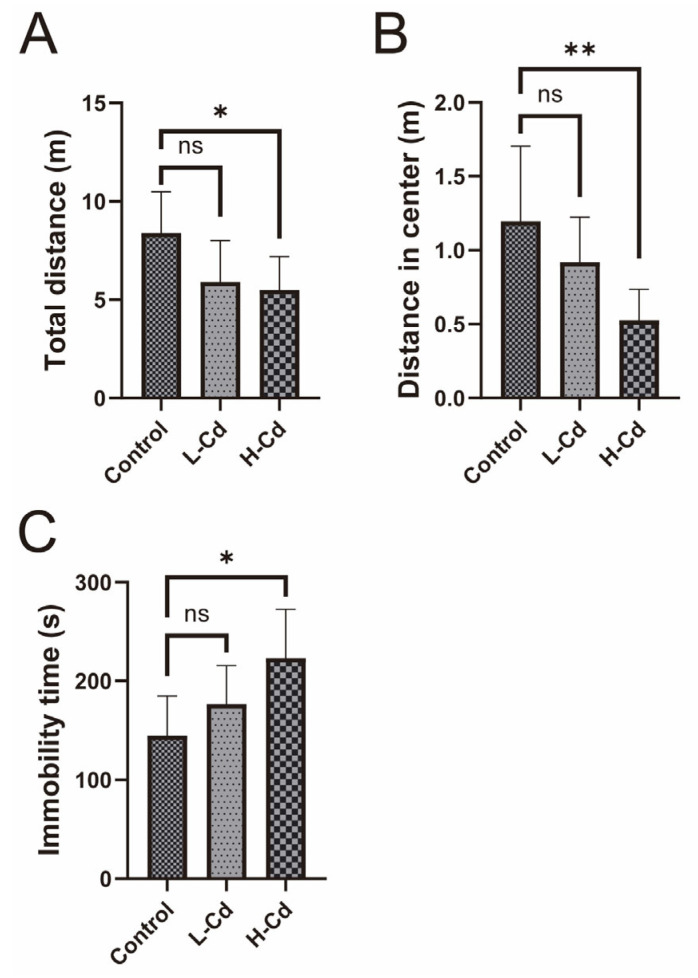
Result of Open Field Test (OFT). (**A**) The total distance of rats. (**B**) The distance in center. (**C**) The immobility time of rats. N = 6 per group, data are presented as mean ± SD, statistical significance was determined by one-way ANOVA followed by Dunnett’s test for multiple comparisons. ns, not significant (*p* ≥ 0.05), * *p* < 0.05, ** *p* < 0.01.

Neurobehavioral performance was evaluated using the open field test, elevated plus maze, and Morris water maze. In the open field test, high-dose cadmium exposure resulted in a significant reduction in total locomotor distance compared with the control group, while the low-dose group showed no significant change (Figure 2A). High-dose cadmium-exposed rats had significantly shorter travel distances in the center area (Figure 2B). High-dose cadmium-exposed rats showed significantly longer immobility time, indicating reduced exploratory behavior (Figure 2C).

**Figure 3:**
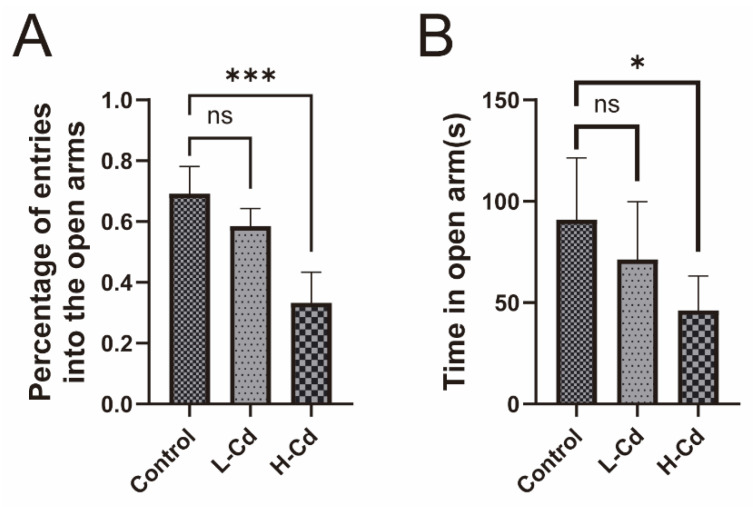
Result of Elevated Plus Maze (EPM): (**A**) Percentage of entries into the open arms. (**B**) Time in open arm. N = 6 per group, data are presented as mean ± SD, statistical significance was determined by one-way ANOVA followed by Dunnett’s test for multiple comparisons. ns, not significant (*p* ≥ 0.05), * *p* < 0.05, *** *p* < 0.001.

In the elevated plus maze, high-dose cadmium exposure significantly decreased both the percentage of entries into the open arms and the time spent in the open arms relative to controls, while the low-dose group showed no significant change (Figure 3A,B), reflecting increased anxiety-like behavior.

### 2.3. Cadmium Exposure Affected Spatial Learning and Memory

**Figure 4:**
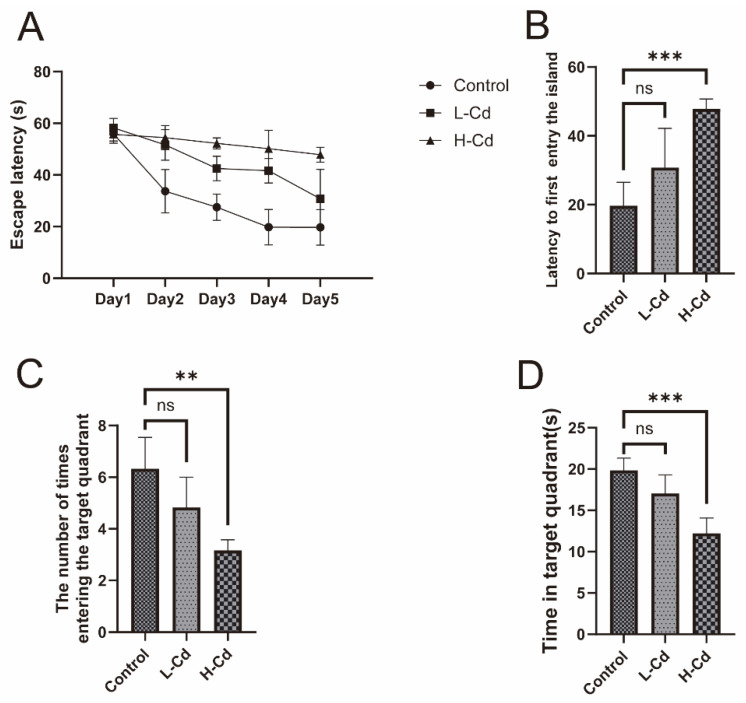
Probe test of Morris Water Maze. (**A**) The escape latency to arrive at the platform. (**B**) Latency to first enter the island. (**C**) The number of times entering the target quadrant. (**D**) Time in target quadrant. N = 6 per group, data are presented as mean ± SD, statistical significance was determined by one-way ANOVA followed by Dunnett’s test for multiple comparisons. ns, not significant (*p* ≥ 0.05), ** *p* < 0.01, *** *p* < 0.001.

Spatial learning and memory were assessed using the Morris water maze. During the acquisition phase, repeated-measures analysis revealed a significant interaction between training day and exposure group for escape latency (Figure 4A). Rats exposed to cadmium showed a slower reduction in escape latency across training days compared with controls, indicating impaired spatial learning.

In the probe trial, high-dose cadmium-exposed rats spent significantly less time in the target quadrant, while the low-dose group showed no significant change (Figure 4D). Latency to first enter the target quadrant was longer than control rats (Figure 4B). The number of entries into the target quadrant was significantly reduced (Figure 4C), demonstrating deficits in spatial memory retention.

### 2.4. Cadmium Exposure Modified Hippocampal Ultrastructure

**Figure 5:**
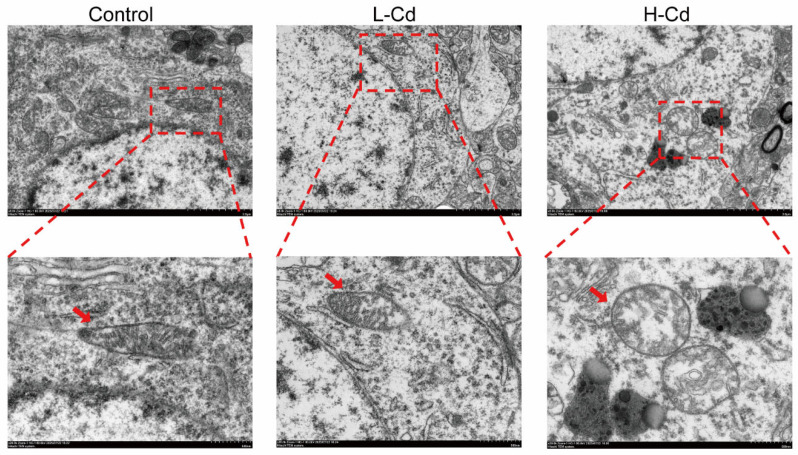
TEM observation of hippocampal neurons (Red arrow indicates impaired mitochondrion).

Transmission electron microscopy (TEM) was used to examine ultrastructural changes in hippocampal neurons. Neurons from control groups exhibited intact nuclear membranes and mitochondria with clearly defined cristae (Figure 5).

In contrast, hippocampal neurons from high Cd group rats displayed pronounced ultrastructural abnormalities, including mitochondrial swelling, disrupted or fragmented cristae, and increased electron-lucent areas within the mitochondrial matrix.

### 2.5. Cadmium Exposure Induced Redox Imbalance and Altered Expression of Disulfidptosis-Related Genes in Hippocampal Tissue

**Figure 6:**
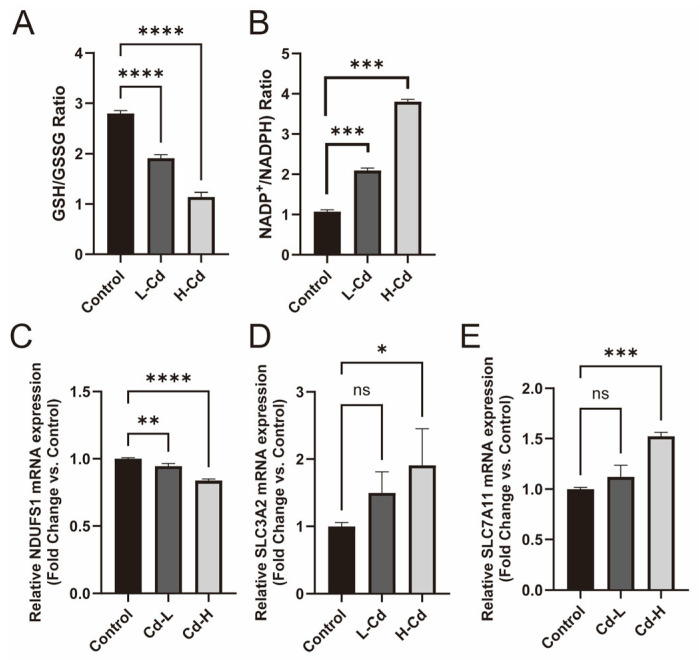
Cadmium Exposure Induced Redox Imbalance and Altered Expression of Disulfidptosis-Related Genes in the Hippocampus. (**A**) GSH/GSSG ratio. (**B**) NADP^+^/NADPH ratio. (**C**) NDUFS1, (**D**) SLC3A2, and (**E**) SLC7A11 were quantified by RT-qPCR and normalized to the corresponding internal control. N = 6, per group, data are presented as mean ± SD, statistical significance was determined by one-way ANOVA followed by Dunnett’s test for multiple comparisons. ns, not significant (*p* ≥ 0.05), * *p* < 0.05, ** *p* < 0.01, *** *p* < 0.001, **** *p* < 0.0001.

To investigate whether cadmium exposure affects redox homeostasis and disulfidptosis-related pathways, redox metabolic indicators and key molecular markers were assessed in hippocampal tissue. Cadmium exposure significantly increased the NADP^+^/NADPH ratio compared with controls ([Control: 1.07 ± 0.044] vs. [High-dose: 3.80 ± 0.059], *p* < 0.05), while the GSH/GSSG ratio was markedly decreased ([Control: 2.80 ± 0.059] vs. [High-dose: 1.14 ± 0.091], *p* < 0.05) (Figure 6A,B), indicating impaired redox balance. Furthermore, cadmium exposure dose-dependently upregulated the mRNA expression of SLC7A11 and SLC3A2, two critical components of the cystine/glutamate antiporter system Xc^−^. Compared with the control group, the high-dose cadmium group showed significant increases in SLC7A11 expression (1.48 ± 0.12-fold, *p* < 0.01) and SLC3A2 expression (1.91 ± 0.55-fold, *p* < 0.05), whereas the low-dose group showed no significant alterations in either gene (Figure 6D,E). In contrast, the expression of NDUFS1, a key mitochondrial complex I subunit, was significantly reduced following high-dose cadmium exposure, decreasing to 0.84 ± 0.01-fold of the control level (*p* < 0.01; Figure 6C). These findings suggest that cadmium-induced redox disruption is accompanied by alterations in disulfidptosis-related molecular pathways in the hippocampus.

## 3. Discussion

To further explore the molecular basis underlying cadmium-induced neurotoxicity, we integrated bioinformatics analysis with experimental validation. By intersecting cadmium-related genes, neurodegenerative disease-related genes, and disulfidptosis-associated genes, seven common genes were identified, including NCKAP1, NDUFS1, NDUFS5, NUBPL, RPN1, SLC3A2, and SLC7A11. Functional enrichment analysis indicated that these genes were mainly involved in mitochondrial respiratory chain function, oxidative phosphorylation, amino acid transport, and redox regulation. Notably, pathways related to neurodegenerative disease and oxidative phosphorylation were significantly enriched, suggesting that disulfidptosis may represent a mechanistic bridge connecting cadmium exposure to neurodegenerative disease. Experimental validation confirmed the altered expression of selected disulfidptosis-related genes (SLC7A11, SLC3A2, and NDUFS1).

In this study, we investigated cadmium-induced neurotoxicity under an environmentally relevant cadmium exposure scenario using an animal model. The results demonstrate that cadmium exposure produced consistent neurobehavioral deficits, hippocampal ultrastructural abnormalities, redox imbalance, and coordinated alterations in molecular markers associated with disulfidptosis. Together, these findings suggest that cadmium-induced neurotoxicity is associated with molecular features consistent with disulfidptosis.

Our findings are consistent with emerging evidence suggesting that disulfidptosis contributes to neuronal injury across diverse neurological disorders. Ye et al. [20] demonstrated in a Parkinson’s disease model that rotenone-induced dopaminergic neurodegeneration was accompanied by molecular and structural features characteristic of disulfidptosis, including increased expression of SLC7A11 and SLC3A2, excessive disulfide bond formation, and activation of the Rac1/WRC/Arp2/3 signaling pathway, ultimately leading to actin cytoskeleton collapse. Similarly, disulfidptosis has been implicated in Alzheimer’s disease pathogenesis, where it may directly or indirectly modulate Aβ accumulation, tau hyperphosphorylation, and oxidative stress [21]. Seizure-induced neuronal glucose deficiency has also been shown to trigger disulfidptosis through abnormal disulfide accumulation [22]. Collectively, these studies suggest that disulfidptosis is not restricted to cancer biology but may also contribute to neuronal injury under diverse pathological conditions, which is consistent with the disulfidptosis-associated molecular alterations observed in the hippocampus of cadmium-exposed rats in the present study.

However, the majority of disulfidptosis research to date has focused on cancer cells—particularly those with high SLC7A11 expression under glucose starvation conditions—where rapid NADPH depletion and cystine accumulation trigger cell death [11]. In contrast, our study demonstrates disulfidptosis-associated molecular alterations under cadmium exposure, a fundamentally different cellular context. This discrepancy raises important questions about the universality of disulfidptosis mechanisms across cell types and suggests that the pathway may be more broadly relevant than originally appreciated [23]. The fact that neurons—which are highly metabolically active and dependent on continuous NADPH regeneration—exhibit disulfidptosis-associated changes under cadmium exposure extends the relevance of this cell death pathway beyond proliferative cancer cells to environmental neurotoxicology [23].

### 3.1. Bioinformatics Analysis Suggests a Link Between Cadmium Exposure, Disulfidptosis, and Neurodegenerative Disease

KEGG enrichment revealed significant enrichment of the ferroptosis pathway. SLC7A11 and SLC3A2 constitute the system Xc^−^ transporter, a central regulator of cystine uptake and cellular redox homeostasis [24]. Emerging evidence indicates that ferroptosis and disulfidptosis share common metabolic regulators, suggesting potential crosstalk between these two forms of regulated cell death [25].

The enrichment of oxidative phosphorylation was mainly driven by NDUFS1, NDUFS5, and NUBPL, which are essential components of mitochondrial Complex I [26,27,28]. Mitochondrial dysfunction and impaired oxidative phosphorylation are well-established mechanisms contributing to neurodegenerative diseases [29].

Interestingly, the NAFLD pathway was also enriched. Recent studies have highlighted a close relationship between disulfidptosis and metabolic dysfunction-associated steatotic liver disease (MASLD) [30], where disturbances in cystine metabolism, redox homeostasis, and NADPH availability contribute to cellular susceptibility to disulfide stress [31].

Retrograde endocannabinoid signaling has been implicated in neuronal communication and dopaminergic regulation, although its precise role in cadmium-induced neurotoxicity requires further investigation [32,33].

These findings suggest that cadmium exposure may contribute to neurodegenerative processes through mitochondrial dysfunction, oxidative stress, and disulfidptosis-related mechanisms.

### 3.2. Cadmium Exposure Induces Neurobehavioral Impairment and Mitochondrial Damage

Cadmium exposure resulted in impaired locomotor activity, increased anxiety-like behavior, and deficits in spatial learning and memory [34]. These functional alterations were accompanied by pronounced mitochondrial ultrastructural damage in hippocampal neurons, characterized by swelling and disruption of cristae [35]. Such mitochondrial abnormalities are consistent with reports describing cadmium-induced mitochondrial dysfunction in neural tissues and support the notion that mitochondrial impairment represents a central component of cadmium neurotoxicity [36]. Notably, the bioinformatics analysis identified significant enrichment of the oxidative phosphorylation pathway and highlighted NDUFS1, NDUFS5, and NUBPL as potential key targets. These genes are closely associated with mitochondrial complex I function, which plays a critical role in ATP production and electron transport [37]. The behavioral performance and mitochondrial morphology observed under cadmium exposure conditions further indicates that redox-related factors may influence the severity of cadmium-induced neural dysfunction.

### 3.3. Cadmium Exposure Promotes a Disulfidptosis-Permissive Redox Environment

Accumulating evidence suggests that NADPH depletion represents a critical metabolic prerequisite for disulfidptosis [38]. Under conditions of elevated cystine uptake, intracellular disulfides continuously accumulate and require reducing equivalents for detoxification [39]. NADPH serves as the major source of reducing power for the regeneration of reduced glutathione and thioredoxin, which are responsible for maintaining protein thiol homeostasis [40]. Consequently, insufficient NADPH availability can promote excessive disulfide accumulation and increase susceptibility to disulfidptosis-associated cellular injury.

In the present study, cadmium exposure not only reduced NADPH availability but also significantly impaired glutathione redox balance. The decreased GSH/GSSG ratio indicates a shift toward a more oxidizing intracellular environment, suggesting that antioxidant defenses were compromised [41]. These findings are consistent with previous reports demonstrating that cadmium induces oxidative stress through disruption of mitochondrial function and antioxidant systems.

Moreover, mitochondrial damage observed in cadmium-exposed rats may further aggravate redox imbalance [42]. Because mitochondrial metabolism is closely coupled to cellular redox homeostasis, impairment of mitochondrial function may exacerbate NADPH depletion and oxidative stress, thereby amplifying susceptibility to disulfide stress [43]. Taken together, these results indicate that cadmium exposure establishes a metabolic and redox environment favorable for the activation of disulfidptosis-associated pathways.

### 3.4. Experimental Validation Supports the Involvement of Disulfidptosis-Related Genes in Cadmium Neurotoxicity

SLC7A11 and SLC3A2 together constitute the heterodimeric cystine/glutamate antiporter system Xc^−^, which mediates cellular cystine uptake and plays a critical role in maintaining intracellular redox homeostasis [44]. Increased expression of these transporters may enhance cystine influx and subsequently increase intracellular disulfide accumulation. Under physiological conditions, excessive disulfides can be reduced by NADPH-dependent antioxidant systems [14]. However, the present study demonstrated significant depletion of NADPH and disruption of glutathione redox balance following cadmium exposure, suggesting that the capacity to detoxify accumulated disulfides was compromised [8]. Such metabolic conditions are considered essential prerequisites for the initiation of disulfidptosis.

In contrast, NDUFS1, NDUFS5, and NUBPL are closely associated with mitochondrial complex I assembly and oxidative phosphorylation. NDUFS1 functions as a core catalytic subunit of complex I, whereas NDUFS5 and NUBPL contribute to complex I stability and biogenesis [26,27,28]. Downregulation of these genes may impair mitochondrial electron transport, reduce ATP production, and exacerbate reactive oxygen species generation. Notably, oxidative phosphorylation was one of the most significantly enriched pathways identified in the KEGG analysis, highlighting the potential contribution of mitochondrial dysfunction to cadmium-induced neurotoxicity.

Interestingly, ferroptosis was also significantly enriched in the bioinformatics analysis. Because SLC7A11 serves as a central regulator of both ferroptosis and disulfidptosis, these findings suggest that multiple forms of redox-dependent cell death may coexist or interact in response to cadmium exposure [45]. Recent studies have proposed that disulfidptosis and ferroptosis share common upstream metabolic regulators, particularly those involved in cystine metabolism and NADPH homeostasis [12].

It is noteworthy that SLC3A2 and SLC7A11 upregulation reached statistical significance only at the high-dose level, while the low-dose group showed a similar but non-significant upward trend. This observation may reflect a threshold-dependent transcriptional response, wherein low-dose cadmium exposure does not sufficiently overwhelm endogenous antioxidant defenses to drive robust SLC3A2 expression [39,46]. Alternatively, the lack of statistical significance in the low-dose group could be attributed to limited statistical power due to the relatively small sample size (n = 6 per group) for detecting modest effect sizes. Nevertheless, the consistent directional trend across doses supports the interpretation that cadmium exposure promotes SLC3A2 expression, and this transcriptional response becomes fully manifest only under higher exposure conditions. Taken together, the coordinated upregulation of SLC7A11/SLC3A2 and downregulation of NDUFS1/NDUFS5/NUBPL, combined with NADPH depletion and glutathione imbalance, supports the existence of a cellular environment permissive for disulfidptosis-like processes. Although direct evidence of cytoskeletal disulfide accumulation and collapse was not assessed in the present study, these findings provide mechanistic support for the hypothesis that disulfidptosis-associated molecular alterations contribute to cadmium-induced neurotoxicity and may represent a potential link between environmental cadmium exposure and neurodegenerative disease-related neurodegeneration.

### 3.5. The Mechanism by Which Neurons Are Highly Susceptible to Sulfhydryl-Mediated Death

Several biological characteristics may render neurons particularly vulnerable to disulfidptosis-associated injury. First, neurons are post-mitotic cells with exceptionally high metabolic demands, reflecting the intense mitochondrial oxidative phosphorylation required to sustain neuronal function [47]. This heavy reliance on mitochondrial respiration makes neurons particularly dependent on continuous NADPH regeneration, as NADPH is essential for maintaining glutathione and thioredoxin in their reduced states [48]. Second, neurons have limited regenerative capacity and cannot readily replace lost cells, making any cell death event—including disulfidptosis—particularly consequential [49]. Third, SLC7A11, a key component of the cystine/glutamate antiporter system Xc^−^, is expressed in neuronal and glial cells and plays an essential role in cystine uptake and redox homeostasis [50]. Under conditions of glucose deficiency or impaired NADPH generation, this high SLC7A11 expression creates a vulnerability: increased cystine uptake combined with insufficient reducing equivalents leads to aberrant disulfide bond formation in cytoskeletal proteins [51]. Fourth, the brain has relatively low levels of metallothionein—a cysteine-rich metal-binding protein that provides cytoprotection against cadmium toxicity—compared with the liver and kidney, limiting its intrinsic detoxification capacity [52]. Collectively, these metabolic and cellular characteristics may increase neuronal vulnerability to disulfidptosis-associated injury under conditions of cadmium-induced redox imbalance.

### 3.6. The Possibility of Cd Indirectly Inducing Brain Cell Damage Through Its Effects on Other Organs Such as the Liver and Kidney

Although the present study primarily investigated the direct effects of cadmium exposure on the hippocampus, cadmium-induced injury in peripheral organs may also indirectly contribute to neurotoxicity through systemic mechanisms. Cadmium preferentially accumulates in the liver and kidneys, the principal target organs of cadmium toxicity, where chronic exposure induces oxidative stress, mitochondrial dysfunction, inflammation, autophagy, and multiple forms of regulated cell death [53]. Dysfunction of these organs may subsequently influence the central nervous system through the liver–brain and kidney–brain axes. For example, hepatic injury may impair systemic glucose and lipid metabolism, antioxidant defenses, and metabolic homeostasis, thereby increasing neuronal susceptibility to oxidative and disulfide stress [9]. Likewise, cadmium-induced nephrotoxicity may reduce the clearance of uremic toxins, inflammatory mediators, and oxidative metabolites, facilitating blood–brain barrier dysfunction and exacerbating neuronal injury [54,55]. Furthermore, accumulating evidence indicates that cadmium-induced hepatic and renal injury promotes the systemic release of pro-inflammatory cytokines, including TNF-α, IL-1β, and IL-6, which can compromise blood–brain barrier integrity, activate microglia, and amplify neuroinflammatory responses [56]. Therefore, cadmium-induced neurotoxicity is likely to result from the combined effects of direct neuronal exposure and indirect systemic toxicity mediated by peripheral organ dysfunction. Although these mechanisms were not directly examined in the present study, they provide an important framework for understanding the multi-organ toxicity of cadmium and warrant further investigation in future mechanistic studies.

### 3.7. Protective Agents Against Cadmium-Induced Neuronal Damage

Given the central role of oxidative stress and redox imbalance in cadmium-induced neurotoxicity, numerous interventions have been investigated to protect neuronal cells. Natural compounds such as sinomenine hydrochloride and quercetin have been shown to alleviate cadmium-induced neuronal injury by enhancing antioxidant defenses, activating the Nrf2/HO-1 or PPARγ signaling pathways, and suppressing oxidative stress and neuroinflammation [57,58]. Likewise, ARA290 exerts neuroprotective effects by reducing oxidative damage, inflammatory cytokine production, and DNA injury, whereas nano-selenium supplementation improves antioxidant capacity and attenuates cadmium-induced cerebellar toxicity [59]. Although these agents effectively mitigate cadmium-induced neuronal injury in experimental models, their clinical application remains limited. More importantly, whether these protective agents directly inhibit disulfidptosis has not yet been investigated. Recent evidence showing that metformin suppresses neuronal disulfidptosis by stabilizing mitochondrial complex I suggests that targeting mitochondrial redox homeostasis may represent a promising therapeutic strategy [60]. Future studies should determine whether established neuroprotective agents can alleviate cadmium-induced neurotoxicity by suppressing disulfidptosis-associated molecular alterations.

Previous studies have primarily attributed cadmium neurotoxicity to oxidative stress, mitochondrial dysfunction, inflammation, and apoptosis, whereas the role of disulfidptosis has remained largely unexplored. By integrating bioinformatics analysis with behavioral assessment, ultrastructural observation, redox metabolite determination, and molecular validation, our study provides evidence that cadmium exposure creates a disulfidptosis-permissive cellular environment characterized by impaired redox homeostasis and altered expression of key regulatory genes. These findings extend the potential relevance of disulfidptosis beyond cancer biology to environmental neurotoxicology and identify SLC7A11, SLC3A2, and NDUFS1 as promising molecular targets for future mechanistic investigation.

### 3.8. Limitations

This study has several limitations. First, although the observed molecular alterations were consistent with disulfidptosis, direct evidence of disulfide bond accumulation and cytoskeletal collapse was not evaluated. Second, only mRNA expression levels were assessed, and further studies are needed to confirm protein-level changes and their functional significance. Third, although bioinformatics analysis suggested a potential link between cadmium exposure, disulfidptosis, and neurodegenerative disease, additional mechanistic studies are required to establish causal relationships. Finally, this study lacks direct measurement of cadmium concentration in blood or hippocampal tissue. While the observed dose-dependent neurobehavioral and molecular alterations strongly suggest cadmium exposure and target tissue accumulation, future studies should incorporate tissue cadmium measurements to establish a complete exposure–dose–response relationship.

## 4. Materials and Methods

### 4.1. Bioinformatics Analysis

To explore the potential role of disulfidptosis in cadmium-associated neurodegenerative disease, genes related to cadmium exposure and neurodegenerative disease were retrieved from the Comparative Toxicogenomics Database (CTD, http://ctdbase.org/). A set of ten reported disulfidptosis-related genes (SLC7A11, SLC3A2, RPN1, NCKAP1, NUBPL, NDUFS1, NDUFS5, LRPPRC, OXSM, and GYS1) [11] was collected from the previous literature. Venn analysis (InteractiVenn, https://www.interactivenn.net/) was performed to identify overlapping genes among cadmium-related genes, neurodegenerative disease-related genes, and disulfidptosis-related genes. GO and KEGG enrichment analyses were performed using the DAVID database (https://davidbioinformatics.nih.gov/), and the enrichment results were visualized using the Bioinformatics online platform (https://www.bioinformatics.com.cn/). Significantly enriched terms were defined as those with an adjusted *p* value < 0.05. Based on the bioinformatics results, overlapping genes were selected for experimental validation in hippocampal tissues from cadmium-exposed rats.

### 4.2. Experimental Animals

A total of 18 healthy adult male Sprague Dawley (SD) rats, weighing 180–220 g, were purchased from Henan Skubes Biotechnology Co., Ltd., China (Anyang, Henan, China), production license number: SCXK (Yu) 2020-0005. All rats were housed individually in standard polypropylene cages with stainless steel wire lids and corn cob bedding, under controlled conditions: ambient temperature of 22 ± 2 °C, relative humidity of 50% ± 10%, and a 12-hour light/dark cycle (light period: 08:00 a.m. to 08:00 p.m.). During the experiment, rats had ad libitum access to standard rodent chow and sterilized drinking water. No mortality were observed in any of the treatment groups during the 30-day exposure period. All experimental procedures were performed in accordance with the national and institutional guidelines for the care and use of laboratory animals, and were approved by the Animal Ethics Committee of Guilin Medical University (Approval No. GLMU-IACUC-202510148).

### 4.3. Experimental Grouping and Administration

After one week of acclimatization feeding, rats were randomly divided into 3 groups (n = 6 per group). The specific grouping and treatments were as follows: Control Group (Control): Administered ddH_2_O daily by gavage. Cadmium Low-Dose Group (Cd-L): Administered a cadmium chloride (CdCl_2_) solution daily by gavage at a dose of 0.036 mg/kg body weight (bw). Cadmium High-Dose Group (Cd-H): Administered a cadmium chloride (CdCl_2_) solution daily by gavage at a dose of 3.6 mg/kg bw. The low-dose cadmium exposure level (0.036 mg/kg bw/day) was selected to model an environmentally relevant human exposure scenario. This dose was derived from a dietary cadmium exposure survey conducted in Guangxi, China, which reported a monthly cadmium intake of approximately 61.78 μg/(kg·BW) among local residents, corresponding to a daily intake of approximately 2 μg/(kg·BW) [61]. To translate this human exposure level to an equivalent rat dose, allometric scaling based on body surface area was applied using the standard conversion factor of 0.018 for humans (70 kg) to rats (200 g), yielding a calculated dose of approximately 0.036 μg/kg/day in rats. A safety factor of 1000 was then applied to account for interspecies differences, inter-individual variability, and the inherent uncertainties in extrapolating from population exposure data to controlled animal studies, resulting in the final low-dose of 0.036 mg/kg/day for oral gavage administration. The high-dose cadmium exposure level (3.6 mg/kg/day) was set at 100 times that of the low dose. This dose was not intended to represent an environmentally relevant exposure level, but rather to serve as a positive reference for establishing a clear dose–response relationship and enabling adequate contrast for evaluating potential threshold effects. Previous studies demonstrating neurobehavioral and neurophysiological alterations following oral administration of approximately 3.5 mg/kg CdCl_2_ in rats [62,63]. Furthermore, studies using oral CdCl_2_ exposure for 30 days have reported hippocampal damage, oxidative stress, and cognitive impairment, supporting the suitability of this exposure paradigm for investigating cadmium-induced neurotoxicity [63]. All administration solutions were freshly prepared using ddH_2_O. A uniform gavage volume of 5 mL/kg bw was used for all treatments, which continued for 30 days. Cadmium chloride (CdCl_2_, CAS No. 10108-64-2, molecular weight 183.32 g/mol) was purchased from Shanghai McLyn Biochemical Technology Co., Ltd. CdCl_2_ is highly soluble in water. All dosing solutions were freshly prepared by dissolving the appropriate amount of CdCl_2_ in ddH_2_O on the day of administration and were thoroughly vortexed to ensure complete dissolution before gavage.

### 4.4. Behavioral Testing

After the 30-day exposure period, behavioral assessments were conducted in the following order: open field test (OFT), elevated plus maze (EPM), and Morris water maze (MWM). To minimize potential carryover effects between tests, animals were allowed to rest for at least 24 h between the OFT and EPM. The MWM was initiated 24 h after completion of the EPM. This testing sequence was selected to progress from less stressful to more demanding behavioral paradigms, thereby reducing the potential influence of prior testing on subsequent behavioral performance. In our study, the behavioral parameters were collected using an automated video-tracking system (ANY-maze), which provides unbiased digital readouts without manual subjective scoring.

A series of behavioral tests were conducted to assess spontaneous locomotor activity, anxiety-like behavior, and spatial learning and memory. All behavioral experiments were performed during the light phase under standardized environmental conditions.

#### 4.4.1. Open Field Test

This test was used to assess spontaneous locomotor activity and exploratory behavior. Each rat was placed individually in the center of a square open field arena (100 cm × 100 cm × 40 cm) and allowed to explore freely for 5 min. A video tracking system recorded the total distance traveled, as well as the time spent and distance moved in the central zone.

#### 4.4.2. Elevated Plus Maze

This test was employed to evaluate anxiety-like behavior. The maze consisted of two open arms and two enclosed arms arranged in a cross shape and elevated 50 cm above the floor. The rat was placed in the central area facing an open arm, and its activity was recorded for 5 min. The number of entries and time spent in the open and closed arms were recorded. The ratio of open arm entries and the ratio of time spent in the open arms were calculated.

#### 4.4.3. Morris Water Maze

This test was utilized to assess spatial learning and memory. The experiment lasted for 6 days, comprising a 5-day navigation training phase and a spatial probe trial on the final day. During the first 5 days, rats underwent 4 training trials per day. In each trial, the rat was placed into the circular pool facing the wall from different starting points, and the time required to find and climb onto a hidden submerged platform (escape latency) was recorded. On the 6th day, the platform was removed. The rat was placed in the water from the quadrant opposite to the original platform location, and the time spent in the target quadrant (where the platform was previously located) and the number of platform crossings were recorded over a 60-s period.

### 4.5. Sample Collection

Following the completion of all behavioral tests, the rats were fasted for 12 h with free access to water before sample collection. All rats were euthanized under deep anesthesia induced by intraperitoneal injection of sodium pentobarbital (40 mg/kg bw). Blood was collected from the abdominal aorta using 5 mL disposable syringes with 21-gauge needles and transferred into EDTA-coated anticoagulant tubes. The blood samples were centrifuged at 3000 rpm and 4 °C for 15 min. The supernatant was collected, aliquoted, and stored at −80 °C in an ultra-low temperature freezer for subsequent analysis.

Following blood collection, rats were rapidly decapitated using sharp surgical scissors. The whole brains were carefully excised and placed onto an ice-cold glass plate moistened with sterile phosphate-buffered saline (PBS, pH 7.4). The hippocampi were bilaterally dissected under a stereomicroscope using fine forceps and surgical blades, following anatomical landmarks (the hippocampal fissure and the ventricular surface). All dissection procedures were completed within 5 min post-decapitation to minimize RNA degradation. Hippocampal tissue was immediately placed into pre-labeled RNase-free cryovials, flash-frozen in liquid nitrogen for 5 min, and then transferred to a −80 °C freezer for long-term storage pending RNA extraction and biochemical assays.

For transmission electron microscopy, a small piece of hippocampal tissue (approximately 1 mm^3^) was immediately fixed in 2.5% glutaraldehyde in 0.1 M phosphate buffer (pH 7.4) at 4 °C for 24 h, then transferred to fresh fixative and stored at 4 °C until further processing. Any remaining tissue was stored at −80 °C as backup.

All cadmium-contaminated biological waste, including animal carcasses, blood-contaminated materials, and cadmium-containing solutions, were collected in designated biohazard containers with clear labels indicating the contents and date of collection. All labeled waste containers were promptly transported to the Animal Experimental Center of Guilin Medical University for centralized disposal.

### 4.6. Detection of Redox Metabolites

To assess the redox status of brain tissue, key coenzymes and the glutathione system were measured. Hippocampal samples were homogenized on ice. The levels of NADP^+^/NADPH and GSH/GSSG were determined strictly according to the protocols of the NADP^+^/NADPH Assay Kit and the GSH/GSSG Assay Kit, respectively. The NADP^+^/NADPH and GSH/GSSG assay kits were purchased from Nanjing Jiancheng Bioengineering Institute (Nanjing, China).

### 4.7. Detection of Gene Expression Levels

To investigate key molecular changes in the pathway associated with disulfidptosis, the mRNA expression levels of related genes were examined. Total RNA was extracted from tissue samples using TRIzol reagent (Sigma, Deutschland, Germany). Using a reverse transcription kit, 1 µg of total RNA was reverse-transcribed into cDNA. This cDNA served as the template for amplification with SYBR Green qPCR Master Mix on a real-time quantitative PCR instrument. The reaction protocol was as follows: initial denaturation at 95 °C for 30 s; followed by 40 cycles of denaturation at 95 °C for 5 s and annealing/extension at 60 °C for 30 s. The relative gene expression levels were calculated using the 2^(^−^ΔΔCt) method.

The mRNA levels were normalized to the internal control β-actin. The primer sequences used for qPCR were listed in Table 1:

### 4.8. Transmission Electron Microscopy Observation

Hippocampus tissue samples fixed in 2.5% glutaraldehyde were rinsed three times with 0.1 M phosphate-buffered saline (PBS, pH 7.4). Subsequently, the samples were post-fixed in 1% osmium tetroxide for 2 h. Following stepwise dehydration using a graded series of ethanol and acetone, the samples were embedded in epoxy resin. Ultrathin sections of 60–80 nm thickness were cut using an ultramicrotome. After double-staining with uranyl acetate and lead citrate, the ultrastructural morphology of mitochondria was observed under a transmission electron microscope. For morphological evaluation, two rats were randomly selected from each group for imaging. The acquired TEM images were independently assessed by two researchers who were blinded to the treatment groups, and the final conclusions were reached by consensus.

### 4.9. Statistical Analysis

All data are presented as the mean ± standard deviation (SD). Statistical analyses were performed using SPSS 28.0 (IBM, Armonk, NY, USA) and GraphPad Prism 8.0 (GraphPad Software, San Diego, CA, USA). Normality and homogeneity of variance were assessed before group comparisons. For comparisons among multiple groups, one-way analysis of variance (ANOVA) was used. When variances were homogeneous, post hoc pairwise comparisons were performed using Tukey’s test; when variances were unequal, Dunnett’s T3 test was applied. Escape latency data from the acquisition phase of the Morris water maze were analyzed using repeated-measures ANOVA, with training day as the within-subject factor and treatment group as the between-subject factor. A two-tailed *p* value < 0.05 was considered statistically significant.

## 5. Conclusions

In summary, cadmium exposure induced neurobehavioral impairment, mitochondrial damage, redox imbalance, and dysregulation of disulfidptosis-related genes in a dose-dependent manner, with the low dose also resulting in significant changes to a few of the endpoint indicators. Integrated bioinformatics and experimental analyses suggest that disulfidptosis-associated molecular alterations—particularly the upregulation of SLC7A11/SLC3A2 and downregulation of NDUFS1—may contribute to cadmium-induced neurotoxicity and provide preliminary mechanistic evidence linking cadmium exposure to disulfidptosis-associated molecular alterations in neurotoxicity.

## Figures and Tables

**Table 1:** RNA primer sequences.

Name	Sequences (5′-3′)
β-actin	Forward: GGCTGTATTCCCCTCCATCG
	Reverse: CCAGTTGGTAACAATGCCATGT
SLC7A11	Forward: GGCACCGTCATCGGATCAG
	Reverse: CTCCACAGGCAGACCAGAAAA
SLC3A2	Forward: TGATGAATGCACCCTTGTACTTG
	Reverse: GCTCCCCAGTGAAAGTGGA
NDUFS1	Forward: AGGATATGTTCGCACAACTGG
	Reverse: TCATGGTAACAGAATCGAGGGA

## Data Availability

The data presented in this study are available from the corresponding author upon reasonable request. Publicly available datasets used for the bioinformatics analysis were obtained from the Comparative Toxicogenomics Database (CTD, http://ctdbase.org/).

## Abbreviations

Cd	Cadmium
CTD	Comparative Toxicogenomics Database
DAVID	Database for Annotation, Visualization and Integrated Discovery
EPM	Elevated Plus Maze
GO	Gene Ontology
GSH	Reduced Glutathione
GSSG	Oxidized Glutathione
KEGG	Kyoto Encyclopedia of Genes and Genomes
MWM	Morris Water Maze
NADPH	Nicotinamide Adenine Dinucleotide Phosphate (Reduced Form)
NADP+	Nicotinamide Adenine Dinucleotide Phosphate (Oxidized Form)
OFT	Open Field Test
ROS	Reactive Oxygen Species
RT-qPCR	Reverse Transcription Quantitative Polymerase Chain Reaction
SD	Sprague Dawley
TEM	Transmission Electron Microscopy
